# Neurocognitive Effects of Preceding Facial Expressions on Perception of Subsequent Emotions

**DOI:** 10.3389/fnbeh.2021.683833

**Published:** 2021-07-30

**Authors:** Shin Ah Kim, Sang Hee Kim

**Affiliations:** Department of Brain and Cognitive Engineering, Korea University, Seoul, South Korea

**Keywords:** emotional conflict, negative emotion, dmPFC, vlPFC, fMRI, functional connectivity

## Abstract

In everyday life, individuals successively and simultaneously encounter multiple stimuli that are emotionally incongruent. Emotional incongruence elicited by preceding stimuli may alter emotional experience with ongoing stimuli. However, the underlying neural mechanisms of the modulatory influence of preceding emotional stimuli on subsequent emotional processing remain unclear. In this study, we examined self-reported and neural responses to negative and neutral pictures whose emotional valence was incongruent with that of preceding images of facial expressions. Twenty-five healthy participants performed an emotional intensity rating task inside a brain scanner. Pictures of negative and neutral scenes appeared, each of which was preceded by a pleasant, neutral, or unpleasant facial expression to elicit a degree of emotional incongruence. Behavioral results showed that emotional incongruence based on preceding facial expressions did not influence ratings of subsequent pictures’ emotional intensity. On the other hand, neuroimaging results revealed greater activation of the right dorsomedial prefrontal cortex (dmPFC) in response to pictures that were more emotionally incongruent with preceding facial expressions. The dmPFC had stronger functional connectivity with the right ventrolateral prefrontal cortex (vlPFC) during the presentation of negative pictures that followed pleasant facial expressions compared to those that followed unpleasant facial expressions. Interestingly, increased functional connectivity of the dmPFC was associated with the reduced modulatory influence of emotional incongruence on the experienced intensity of negative emotions. These results indicate that functional connectivity of the dmPFC contributes to the resolution of emotional incongruence, reducing the emotion modulation effect of preceding information on subsequent emotional processes.

## Introduction

In everyday life, individuals encounter multiple stimuli that are incongruent in emotional valence, both successively and simultaneously. For example, certain facial expressions might be emotionally incongruent with current or subsequent behavioral actions. When an emotional stimulus follows or occurs simultaneously with an emotionally incongruent stimulus, emotional conflict emerges. Previous research has determined that emotional conflict can interfere with goal-oriented cognitive processes (Etkin et al., 2011; Rahm et al., 2013).

Previous studies have investigated the neurocognitive mechanisms underlying emotional conflict by simultaneously presenting task-relevant information and emotionally incongruent distractors (Etkin et al., 2006; Egner et al., 2008; Kotz et al., 2015). Behaviorally, recognizing emotions conveyed by facial expressions tends to be slowed by emotionally incongruent distractor words, such as “happy” or “fear” written on pictures fearful or happy expressions, respectively (Etkin et al., 2006; Egner et al., 2008). Neuroimaging studies have implicated the dorsomedial prefrontal cortex (dmPFC; Rahm et al., 2013; Kotz et al., 2015) and dorsal anterior cingulate cortex (dACC; Etkin et al., 2011; Torres-Quesada et al., 2014) in the detection of emotional conflict and the rostral anterior cingulate cortex (rACC) in adaptation to emotional conflict (Egner et al., 2008; Etkin et al., 2011; Rahm et al., 2013). Related studies have implicated the ventrolateral prefrontal cortex (vlPFC) in regulating emotional interference in various contexts (Aron et al., 2004; Dolcos et al., 2006; Hooker and Knight, 2006; Jonides and Nee, 2006; Roelofs et al., 2009). Hence, the rACC and vlPFC seem to play critical roles in the resolution of emotional conflict (Feng et al., 2018). Furthermore, a previous study has shown functional connectivity between dACC activation in incongruent trials and rACC activation in subsequent incongruent trials (Etkin et al., 2006), which suggests that brain regions associated with conflict detection and resolution are functionally connected.

Additional studies have addressed the detection and resolution of emotional conflict with respect to emotional information presented simultaneously (Ahmed and Sebastian, 2020; Hassel et al., 2020). Fewer studies have investigated the neurocognitive mechanisms of emotional conflict elicited by preceding stimuli that are emotionally incongruent with subsequent stimuli (Werheid et al., 2005; Rohr et al., 2012). Moreover, most extant studies have examined the effects of emotional conflict on cognitive performance, such as affect categorization (Werheid et al., 2005; Zhang et al., 2010; Rohr et al., 2012). Less attention has been paid to changes in individuals’ experienced emotions and associated neural responses. In one of the few studies examining the effect of preceding stimuli on experienced emotions and neural responses to subsequent incongruent stimuli, Leknes et al. (2013) showed that the affective valence relative to the preceding emotional information alters individuals’ perceptions of pain. Specifically, in their study, participants received thermal pain stimulation following a cue signaling the intensity of the pain. Participants reported reduced pain intensity when a moderate pain followed a cue that signaled intense pain relative to a cue that signaled innocuous heat. Furthermore, the same comparison revealed attenuated skin conductance responses and activity in pain network brain regions such as the insula and dACC (Leknes et al., 2013). This demonstrates clearly that expectations formed by a preceding cue influence the subsequent experience of pain; however, less is known about whether such an effect manifests in the absence of an explicit induction of expectations for the valence of subsequent stimuli and in the absence of external physical sensation.

In the present study, we attempted to examine neural correlates of emotional conflict elicited by the difference in emotional valence between consecutively presented emotional information. Further, we examined how emotional conflict alters the subjective experience of negative emotions. Healthy young men and women participated in a functional magnetic resonance imaging (fMRI) study. We asked participants to rate the emotional intensity for a series of pictures depicting negative and neutral scenes, which were preceded by facial expressions of pleasant, neutral, and unpleasant emotions. We calculated differences in emotional valence between the preceding facial expressions and subsequent pictures as indices of emotional conflict and entered them into a parametric modulation analysis to reveal brain regions where the activity was correlated linearly with the degree of emotional conflict. We also performed functional connectivity analysis to determine whether the brain regions associated with emotional conflict were functionally connected with those associated with conflict resolution. We expected that the experience of emotional intensity in response to negative pictures would be accentuated by preceding emotionally incongruent facial expressions relative to emotionally congruent facial expressions. We also expected that parametric analysis of fMRI data would reveal activation in the dmPFC and dACC, as they are implicated in emotional conflict detection (Etkin et al., 2011; Kotz et al., 2015). Additionally, we expected that the brain regions related to emotional conflict detection would be functionally connected with the regions associated with conflict resolution such as the rACC and vlPFC (Dolcos et al., 2006; Rahm et al., 2013).

## Materials and Methods

### Participants

A total of 25 right-handed college students (13 males, mean age = 23.38 ± 2.36; 12 females, mean age = 22.25 ± 1.96), without previous or current neurological and psychiatric illnesses, were recruited for this study. All of the students provided their written informed consent prior to their participation. This study was approved by the ethics committee of Korea University and performed in accordance with the Declaration of Helsinki. In addition, all of the participants were monetarily compensated for their time. However, one participant was excluded from the analyses, due to the failure to maintain attention during the task.

### Materials and Task

As preceding emotional information, photographs of facial expressions were selected from the Korea University Facial Expression Database (Lee et al., 2012). These expressions were created by the method-acting protocol, in which the actors were asked to imagine themselves in everyday scenarios eliciting pleasant, neutral, or unpleasant emotions. Their expressions were then edited into 2-second-long video clips, showing dynamically changing facial expressions from a neutral to a targeted expression. In total, the facial expressions included 16 pleasant (valence *M* = 5.75, SD = 0.82; arousal *M* = 4.92, SD = 0.72), 16 unpleasant (valence *M* = 2.17, SD = 0.45; arousal *M* = 4.72, SD = 0.54), and 16 neutral emotions (valence *M* = 3.61, SD = 0.33; arousal *M* = 2.83, SD = 0.38), all of which were rated on a seven-point Likert scale ranging from 1 (valence: very unpleasant; arousal: not arousing) to 7 (valence: very pleasant; arousal: strongly arousing).

Based on the normative ratings of valence (i.e., 1 = very unpleasant to 7 = very pleasant) and arousal (1 = not arousing to 7 = strongly arousing), 48 negative (valence *M* = 2.68, SD = 0.44; arousal *M* = 4.60, SD = 0.62) and 48 neutral pictures (valence *M* = 4.30, SD = 0.28; arousal *M* = 3.24, SD = 0.57) were selected from the Korea University Affective Picture System (Kim, 2012). The pictures in each emotional category were then divided into three sets of 16 pictures (with matching levels of emotional valence and arousal) and assigned to one of three facial expression conditions: pleasant, unpleasant, or neutral. In addition, the assignment of the picture sets to each facial expression condition was counterbalanced across the participants.

**Figure 1:**
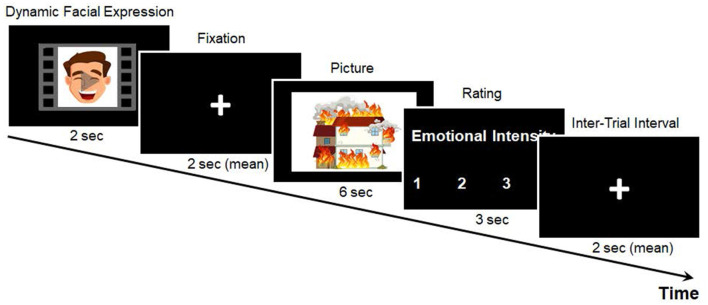
Example of the emotional intensity rating task. Example stimuli are animated for demonstration purposes.

Each trial of the emotional intensity rating task began with a pleasant, neutral, or unpleasant facial expression, which appeared on the screen for 2 s, followed by a fixation with a variable duration (1.5, 2, or 2.5 s). Next, either a negative or a neutral picture was presented for 6 s. After the picture disappeared, a rating scale of 1 (no or weak arousal) to 4 (strong arousal) was presented for 3 s, during which the participants rated their levels of emotional intensity by pressing the corresponding button. As shown in Figure 1, a fixation cross of variable duration (1, 2, or 3 s) was presented before each new trial.

### Procedure

After signing the written consent form, the participants completed the Beck Anxiety Inventory (BAI; Beck et al., 1988) and the Beck Depression Inventory-II (BDI-II; Beck et al., 1996) in order to measure their levels of anxiety and depression. The means and standard deviations for the BAI and BDI were 5.92 ± 4.38 and 8.08 ± 5.35, respectively. Then, before going into the brain scanner, they received instructions regarding the emotional intensity rating task and completed three practice trials, with facial expressions and picture stimuli that were not used in the actual task. After the participants performed the task in the scanner, they were debriefed and compensated for their time.

### Imaging Data Acquisition

All of the images were obtained at the Korea University Brain Imaging Center using a 3T Siemens Trio scanner (Siemens Medical Solutions, Germany). A high-resolution T_1_-weighed whole-brain anatomical scan (1 mm^3^ voxel resolution, MPRAGE) was acquired prior to the functional imaging. Overall, the functional brain images during the task were acquired in 36 axial slices using an echo planar imaging pulse sequence, with a TR of 2,000 ms, a TE of 30 ms, a flip angle of 90°, a field of view of 240 × 240 mm^2^, a matrix size of 64 × 64, and a slice thickness of 4 mm with no gap. The stimuli were presented on a computer screen using fMRI-compatible video goggles (Nordic Neurolab, Bergen, Norway). All of the responses were made via a fiber-optic button box (Current Designs, Philadelphia, PA, USA).

### Imaging Data Analysis

Image preprocessing and statistical analyses were performed using SPM12^1^. After performing a timing correction for interleaved slice acquisition, the functional images were realigned to the first volume to correct for head motion and spatially normalized to a standard stereotaxic space (Montreal Neurological Institute, MNI) implemented in SPM12. They were also resampled at a voxel resolution of 3 mm × 3 mm × 3 mm, and spatially smoothed using a Gaussian kernel, with a full-width at half-maximum (FWHM) of 6 mm. In addition, the realignment parameters were inspected to identify the participants with excessive head movements (translation > 2 mm, rotation > 2 degrees), after which one participant with more than 2 mm of head motion was excluded from the neuroimaging analysis.

The functional image analyses were constrained to the brain regions that showed prior evidence of involvement in emotional conflict processing (Braunstein et al., 2017). For this purpose, a structurally defined inclusive mask was created using the Wake Forest University PickAtlas Tool (Maldjian et al., 2003), with the automated anatomical labeling atlas (AAL: Tzourio-Mazoyer et al., 2002). The following brain regions were bilaterally included in the mask: the inferior frontal gyri, the orbitofrontal cortex, the superior medial frontal gyri, and the anterior cingulate gyri.

#### Parametric Modulation Analysis

A first-level general linear model was created for each individual to identify the brain regions whose activity was keenly associated with the degree of emotional conflict between the preceding facial expressions and the subsequent pictures. The level of incongruence in emotional valence between the facial expressions and pictures was calculated as an index of emotional conflict. Specifically, the parametric modulator was assigned to each condition (as an integer) in accordance with the degree to which the picture was more negatively valenced than the preceding facial expression. In this regard, for pleasant facial expressions that preceded negative pictures (PleasantFace-NegPic), the parametric modulator was 2; for pleasant facial expressions that preceded neutral pictures (PleasantFace_NeuPic) or for neutral facial expressions that preceded negative pictures (NeutralFace_NegPic), the parametric modulator was 1; for unpleasant facial expressions that preceded negative pictures (UnpleasantFace_NegPic) or for neutral facial expressions that preceded neutral pictures (NeutralFace_NeuPic), the parametric modulator was 0; and for unpleasant facial expressions that preceded neutral pictures (UnpleasantFace_NeuPic), the parametric modulator was −1.

The blood-oxygen-level-dependent (BOLD) responses during picture presentation (6 s from picture onset) were modeled as events, along with the parameter convolved with the canonical hemodynamic response function and using a general linear model in SPM12. To control for the potential residual effect of the facial expressions’ emotions on the BOLD responses during picture presentation, the face presentation events (pleasant, neutral, unpleasant faces, respectively), the rating events, and the realign parameters were entered into a general linear model, as regressors of no interest.

Moreover, the parametric maps of each participant were included in the second-level random effects analyses, after which one-sample *t*-tests were conducted to generate statistical inferences. Any corrections for the multiple statistical comparisons were estimated using Monte Carlo simulations conducted in the AFNI program 3dClustSim (ver. 20.3.00), with an initial cluster-forming single-voxel threshold of *p* < 0.005 (uncorrected) within the gray matter inclusive mask (thresholded at 40% intensity), which yielded a minimum cluster size threshold of 41 contiguous voxels to achieve a cluster-wise threshold of *p* < 0.05.

#### Functional Connectivity Analysis

In order to identify brain regions functionally associated with neural correlates of emotional conflict, a psychophysiological interaction (PPI) analysis was conducted for the conflict condition with the greatest incongruence parameter relative to the corresponding non-conflict condition (PleasantFace-NegPic > UnpleasantFace-NegPic). This PPI analysis used a 6 mm radius sphere, centered on the peak of the dmPFC cluster in the parametric modulation analysis, as the source region. For the PPI contrast, time-series data from the dmPFC source region for each participant was extracted and PPI regressors were generated (i.e., the time-course of the activity in the seed region modulated by the psychological variable). Then, a first-level general linear model was estimated for each participant, with the following regressors: (1) the time-course of the activity in the dmPFC region; (2) the psychological contrast (PleasantFace-NegPic > UnpleasantFace-NegPic); (3) the interaction term (dmPFC activity × contrast weight); and (4) the realign parameters. Moreover, these contrasts were entered into the group level analyses by way of one-sample *t*-tests for statistical inferences. In this case, any corrections for the multiple statistical comparisons were estimated using Monte Carlo simulations conducted in the AFNI program 3dClustSim (ver. 20.3.00), with an initial cluster-forming single-voxel threshold of *p* < 0.005 (uncorrected) within the gray matter inclusive mask (thresholded at 40% intensity), which yielded a minimum cluster size threshold of 36 contiguous voxels to achieve a cluster-wise threshold of *p* < 0.05.

#### Correlation Analysis

We examined whether the functional connectivity of the dmPFC was associated with individual differences in perceived emotional intensity modulated by emotional conflict. We conducted a correlation analysis between dmPFC functional connectivity parameters (i.e., first eigenvariates) extracted from the contrast of (PleasantFace-NegPic > UnpleasantFace-NegPic) and changes in emotional intensity ratings for negative pictures calculated from the same condition contrast.

## Results

### Behavioral Results

**Figure 2:**
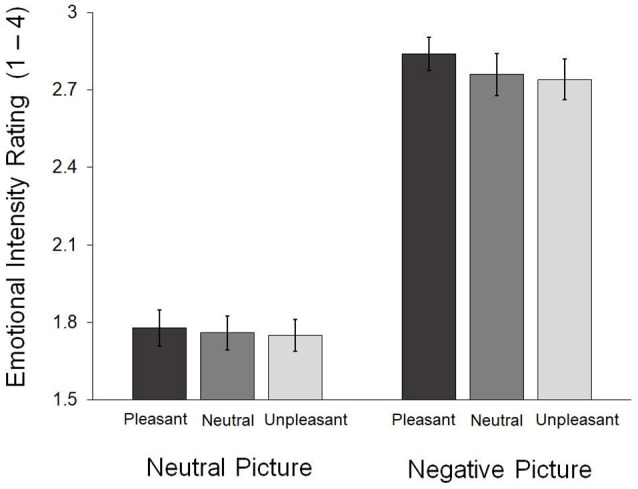
Emotional intensity ratings obtained during the in-scanner task. The error bars indicate the standard errors of the means.

Figure 2 presents the emotional intensity reported in each condition. A repeated 3 (face: pleasant, neutral, unpleasant) × 2 (picture: neutral, negative) analysis of variance (ANOVA) was conducted on the behavioral ratings of emotional intensity. The main effect of picture was significant, *F*_(1,23)_ = 188.94, *p* < 0.001. Negative pictures (*M* = 2.78, SD = 0.34) were rated with greater intensity than the neutral ones (*M* = 1.76, SD = 0.29). We found no main effect of face, *F*_(2,46)_ = 1.81, *p* = 0.175, and no significant interaction, *F*_(2,46)_ = 0.78, *p* = 0.467 (Figure 2).

### Neuroimaging Results

#### Neural Correlates of Emotional Conflict

**Figure 3:**
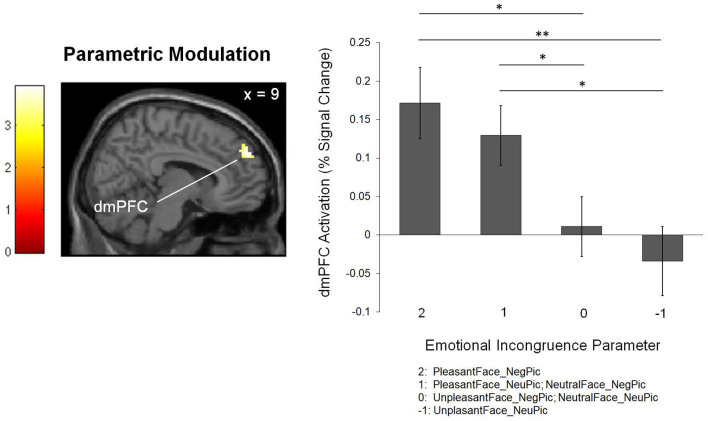
The brain region associated with the level of emotional incongruence. The bar graph shows the percent signal changes in the corresponding region. ^*^p < 0.05, ^**^p < 0.01.

The parametric modulation analysis identified the right dmPFC (BA 9; *x* = 9, *y* = 53, *z* = 57; *k* = 49; *z* = 3.38) as an area in which activity increased as a function of emotional incongruence experienced while processing pictures (Figure 3).

#### Functional Connectivity of the dmPFC

**Figure 4:**
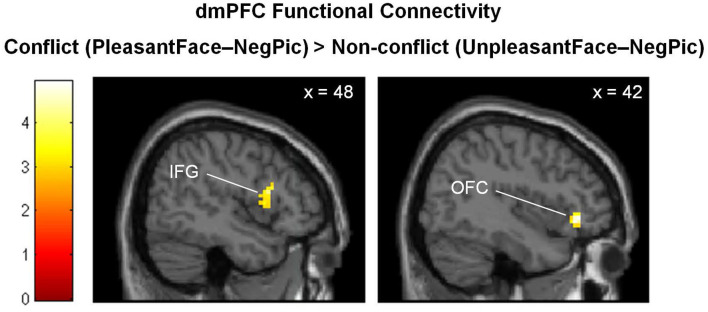
Brain regions showing stronger functional connectivity with the dmPFC in the emotionally incongruent condition, as compared with the emotionally congruent condition. dmPFC, dorsomedial prefrontal cortex; IFG, inferior frontal gyrus; OFC, orbitofrontal cortex.

The PPI analysis revealed increased functional connectivity of the dmPFC with the right orbitofrontal cortex (OFC; BA 47; *x* = 42, *y* = 35, *z* = −5; *k* = 45; *Z* = 4.00) and right inferior frontal gyrus (IFG; BA 48; *x* = 48. *y* = 14, *z* = 16; *k* = 61; *Z* = 3.46) when negative pictures were preceded by pleasant facial expressions relative to unpleasant facial expressions (Figure 4).

#### Correlation Between Functional Connectivity of the dmPFC and Emotional Experience

**Figure 5:**
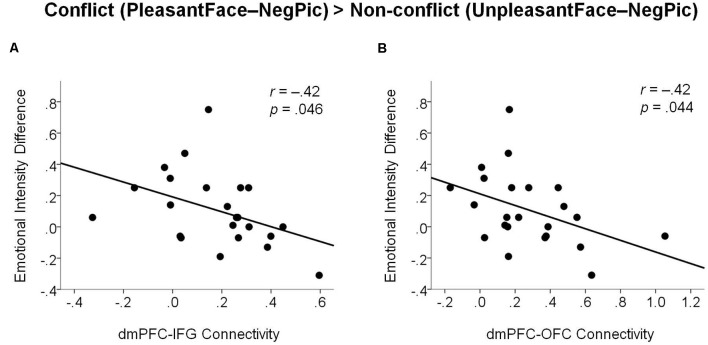
Scatter plots illustrating negative associations between changes in emotional intensity ratings by emotional incongruency and dmPFC functional connectivity with the right IFG **(A)** and with the right OFC **(B)**. dmPFC, dorsomedial prefrontal cortex; IFG, inferior frontal gyrus; OFC, orbitofrontal cortex.

The correlation analysis revealed that the functional connectivity of the dmPFC with both the right IFG (Figure 5A; *r* = −0.42, *p* = 0.044) and OFC (Figure 5B; *r* = −0.42, *p* = 0.046) was negatively correlated with the modulatory increase in negative emotional intensity by emotional conflict. That is, with greater functional connectivity of the dmPFC, emotional incongruence influenced less the intensity of negative emotions experienced by the participants.

## Discussion

The present study examined how experiential and neural responses to negative pictures are influenced by preceding emotionally incongruent facial expressions. As expected, we found that activation in the right dmPFC increased with the level of emotional incongruence between the pictures and the preceding facial expressions. The right dmPFC exhibited greater positive functional connectivity with the right vlPFC (including both the IFG and OFC) for negative pictures preceded by emotionally incongruent pleasant facial expressions than for those preceded by congruent unpleasant facial expressions. The increased functional connectivity between the dmPFC and the vlPFC was negatively correlated with the modulatory increase in experienced negative emotions due to emotional incongruence.

Previous literature on emotional conflict has consistently reported that conflict detection employs the dmPFC and dACC (Egner et al., 2008; Etkin et al., 2011; Rahm et al., 2013; Torres-Quesada et al., 2014; Kotz et al., 2015). Thus, the parametric modulation of right dmPFC activity by emotional incongruence appears to be associated with the detection of emotional conflict between the pictures and prior facial expressions. Alternatively, but not exclusively, the modulation of dmPFC activity by emotional conflict may also relate to expectancy violation (Somerville et al., 2006; Cloutier et al., 2011), although in the current study the preceding emotional stimuli did not cue participants to expect specific subsequent stimuli. That is, facial expressions can generate expectations of emotional information (Schmidt and Cohn, 2001; Dannlowski et al., 2007), and these expectations may have been violated when subsequently presented pictures were emotionally incongruent. The detection of emotional conflict and expectancy violation might share overlapping neurocognitive mechanisms, which warrants further study.

Our results showing increased functional connectivity of the right dmPFC with the right vlPFC in the emotional conflict condition is in line with previous suggestions that the dmPFC sends signals to the vlPFC to control interference from preceding emotional information (Aron et al., 2004; Hooker and Knight, 2006). The vlPFC is the core neural system responsible for the inhibition of goal-irrelevant emotional responses (Aron et al., 2004; Dolcos et al., 2006; Hooker and Knight, 2006; Roelofs et al., 2009). Emotionally incongruent stimuli may generate conflicting response tendencies (Bartholow et al., 2009; Goerlich et al., 2012), and the resolution of the conflicting responses may be achieved by inhibiting the response tendency induced by the preceding emotional stimuli. The correlation result showing that increased functional connectivity of the dmPFC with the vlPFC during conflicting trials was associated with the reduced modulatory influence of emotional incongruence on the subjective experience of emotional intensity for negative pictures emphasizes the importance of functional communication between brain regions responsible for conflict detection and resolution.

It is notable that participants’ task was to rate the emotional intensity of negative and neutral scenic pictures; there was no explicit requirement to resolve emotional conflict while viewing the pictures, which is unlike previous studies in which successful task performance depended on the resolution of the conflict between the target and distractors. Therefore, the current study extends previous research by showing that the neural mechanisms involved in perception and resolution of emotional conflict are similar, regardless of the presence of explicit task goals.

Although our results are revealing, several limitations should be noted. First, contrary to our expectations, emotional conflict elicited by the preceding facial expressions did not influence the self-reported emotional intensity ratings of the pictures. We had participants assign emotional intensity ratings on a scale from 1–4 to minimize difficulties in their button responses while inside the scanner. This may have limited the ability to detect changes in perceived emotional intensity related to our manipulation of the preceding incongruent facial expressions. Considering that there was a numerical trend of linear increase in emotional intensity ratings from the non-conflict condition to the conflict condition for the negative pictures, the limited scale may not have reflected the effect sufficiently. Second, parametric modulation analysis of fMRI data assumes that the degree of emotional conflict elicited by preceding incongruent stimuli is encoded linearly in neural systems. There may be neural systems that encode emotional conflict nonlinearly, which we did not investigate in the current study. Third, our results might be confounded by individual differences in participants’ physical and mental states related to everyday life, including sleep deprivation, caffeine intake, and glucose level, as they have been shown to affect experiential and neural response to emotional stimuli (Schöpf et al., 2013; Cote et al., 2014; Giles et al., 2018). This warrants future studies with larger sample sizes to address the contribution of these factors that might influence emotional processing.

Overall, the findings contribute to our understanding of the neurocognitive mechanisms underlying the perception of conflict in emotional valence between stimuli presented consecutively. We have revealed the roles of the dmPFC and right vlPFC in the detection and resolution of emotional conflict when there is no explicit requirement to resolve such conflict. It is expected that future research will follow up on the present findings and attempt to provide further information about the diverse forms of neurocognitive mechanisms underlying the diverse forms of emotional conflicts encountered in everyday life.

## Data Availability Statement

The original contributions presented in the study are included in the article, further inquiries can be directed to the corresponding author.

## Ethics Statement

The studies involving human participants were reviewed and approved by Ethics Committee of Korea University. The patients/participants provided their written informed consent to participate in this study.

## Author Contributions

SAK designed the study, collected data, analyzed and interpreted data, and wrote the first draft. SHK designed the study, analyzed and interpreted data, and critically revised the manuscript. All authors contributed to the article and approved the submitted version.

## Conflict of Interest

The authors declare that the research was conducted in the absence of any commercial or financial relationships that could be construed as a potential conflict of interest.

## Publisher’s Note

All claims expressed in this article are solely those of the authors and do not necessarily represent those of their affiliated organizations, or those of the publisher, the editors and the reviewers. Any product that may be evaluated in this article, or claim that may be made by its manufacturer, is not guaranteed or endorsed by the publisher.

## Footnotes

^1^ http://www.fil.ion.ucl.ac.uk/spm
